# A feasibility study of the Dynamic Phantom scanner for quality assurance of beam profiles at various gantry angles

**DOI:** 10.1120/jacmp.v6i2.2081

**Published:** 2005-05-21

**Authors:** Yunkai Zhang, Wen C. Hsi, James C.H. Chu, Damian B. Bernard, Ross A. Abrams

**Affiliations:** ^1^ Departments of Medical Physics and Radiation Oncology Rush University Medical Center 1653 West Congress Parkway Chicago Illinois 60612 U.S.A.

**Keywords:** Dynamic Phantom, profile scanner, quality assurance

## Abstract

The effect of gantry rotation on beam profiles of photon and electron beams is an important issue in quality assurance for radiotherapy. To address variations in the profiles of photon and electron beams at different gantry angles, a Dynamic Phantom scanner composed of a $20\times 12\times {6\;\text{cm}}^{3}$ scanning Lucite block was designed as a cross‐beam‐profile scanner. To our knowledge, differences between scanned profiles acquired at different gantry angles with a small size Lucite block and those acquired a full‐size $\left(60\times 60\times {50\;\text{cm}}^{3}\right)$ water phantom have not been previously investigated. We therefore performed a feasibility study for a first prototype Dynamic Phantom scanner without a gantry attachment mount. Radiation beams from a Varian LINAC 21EX and 2100C were used. Photon beams (6 MV and 18 MV) were shaped by either collimator jaws or a Varian 120 Multileaf (MLC) collimator, and electron beams (6 MeV, 12 MeV, and 20 MeV) were shaped by a treatment cone. To investigate the effect on profiles by using a Lucite block, a quantitative comparison of scanned profiles with the Dynamic Phantom and a full‐size water phantom was first performed at a 0° gantry angle for both photon and electron beams. For photon beam profiles defined by jaws at 1.0 cm and 5.0 cm depths of Lucite (i.e., at 1.1 cm and 5.7 cm depth of water), a good agreement (less than 1% variation) inside the field edge was observed between profiles scanned with the Dynamic Phantom and with a water phantom. The use of Lucite in the Dynamic Phantom resulted in reduced penumbra width (about 0.5 mm out of 5 mm to 8 mm) and reduced (1% to 2%) scatter dose beyond the field edges for both 6 MV and 18 MV beams, compared with the water phantom scanner. For profiles of the MLC‐shaped 6 MV photon beam, a similar agreement was observed. For profiles of electron beams scanned at 2.9 cm depth of Lucite (i.e., at 3.3 cm depth of water), larger disagreements in profiles (3% to 4%) and penumbra width (3 mm to 4 mm out of 12 mm) were observed. Additional profiles with the gantry at 90° and 270° were performed for both MLC‐ and jaw‐shaped photon beams and electron beams to evaluate the effect of gantry rotation. General good agreement is seen (less than 1 % variation) at all field sizes for collimator‐shaped 6 MV and 18 MV photon beams. Similar variations observed for MLC‐shaped photon beams indicate that the uncertainty in MLC position is similar to that for the collimator jaws. We conclude that the Dynamic Phantom scanner is a useful device for the routine quality assurance on beam profiles of photon beams and for constancy check on electron beams at various gantry angles. Caution should be taken when using this device to acquire basic electron dosimetry data.

PACS number: 87.53.‐j

## I. INTRODUCTION

There are many advantages associated with the use of a multileaf collimator^(1)^ (MLC), including enhanced precision and ease of dose conformality to designated targets, and protection of critical normal organs and tissues for both 3D conformal and intensity‐modulated radiation therapy. However, a comprehensive process of quality assurance (QA) for the MLC has not yet been fully established due to the complexity of this task. By dividing the MLC QA process into routine LINAC‐based and patient‐specific components, the challenge of MLC QA is more readily approached. During patient‐specific dosimetric verification, the calculated dose distribution for each treatment field with reassigned 0° gantry angle can be verified against one measured from film or MAPCHECK, a diode‐based dose measurement device available in many institutions, including our own. In this verification approach, one assumes that dose profiles and output factors for each treatment field do not vary with gantry angle. One problem associated with this approach is that it does not account for gravitational pulling effects on an individual leaf's location as the gantry angle varies. Therefore, it is essential to consider and measure the gantry‐angle‐dependent dosimetric variation.

Traditionally, films and the water tank scanner are used to measure profiles. Using film dosimetry, many factors need to be considered to obtain accurate relative dose distributions. When using a water‐tank scanner at nonzero gantry angles, one faces an unavoidable setup complexity with a sealed and reduced size water tank that may introduce measurement errors. Therefore, a Dynamic Phantom consisting of a $20\times 12\times {6\;\text{cm}}^{3}$ Lucite block was designed as a cross‐beam‐profile scanner by Advanced Radiation Measurements Inc.^(2)^ With a gantry attachment mount, required radiotherapy beam profiles at different gantry angles for routine beam profile QA can be performed with an easy setup. Due to its finite size, the Dynamic Phantom may underestimate the scatter dose near and outside of the field edge, especially for large field sizes. However, based on Bragg‐Gray theorem, the main contribution to the measured dose comes from secondary electrons from the small volume surrounding the radiation detector in a charged‐particle equilibrium condition. Therefore, profiles measured at a certain depth in Lucite with the Dynamic Phantom should be similar to those measured at an effective depth in water with a water phantom. The effective depths of scanned profiles with a water tank scanner were calculated according to the electron density of Lucite. For a feasibility study on a first prototype Dynamic Phantom scanner, quantitative comparison of scanned profiles for 5 cm, 10 cm, 15 cm, 20 cm, and 30 cm field sizes with the Dynamic Phantom at 1.0 cm and 5.0 cm depths in Lucite and a full‐size water phantom at 1.1 cm and 5.7 cm effective depth were performed at a 0° gantry angle for the 6 MV and 18 MV photon beams. For a constancy check on the effect of gantry rotation, additional scanned profiles with the gantry at 90° and 270° with the same field sizes at 1.0 cm and 5.0 cm depths in Lucite were also performed with 6 MV and 18 MV photon beams. To examine the use of the Dynamic Phantom with electron beams, measurements of various field sizes (10 cm, 15 cm, and 20 cm) at different depths with 6 MeV, 12 MeV, and 20 MeV electrons were performed at 0°, 90°, and 270° gantry angles. Based on quantitative comparisons of photon/electron beam profiles at different gantry angles, we evaluate the suitability of the Dynamic Phantom scanner to determine the effect of gantry rotation on beam profiles in routine QA.

## II. MATERIALS AND METHODS

### A. Dynamic Phantom and water tank scanners

**Figure 1:**
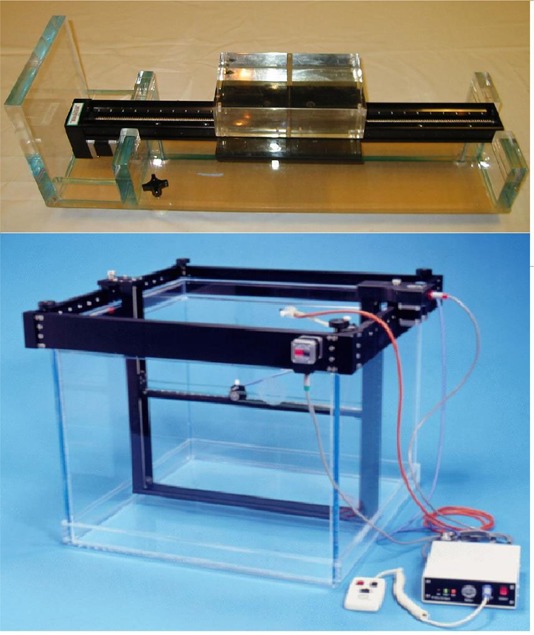
The Dynamic Phantom and water‐tank scanners used in this study

The Dynamic Phantom scanner used in this study is shown in the top panel of Fig. 1. A Lucite block is moved along a threaded steel rod, driven by a stepping motor controlled by scanning software. The maximum scan range is 45 cm with a 1 mm scanning interval. The electron density of Lucite^(3)^ is 1.137 with respect to water, and its physical density is 1.1364 g/cm^3^. An insertion tunnel at one side of the Lucite slab is used to place an ionization chamber for measurements. The depth of each scanned profile includes the thickness of Lucite in the beam path of the detector inserting slab.

The water‐tank scanner used in this study has 3D scanning capability as shown in the bottom panel of Fig. 1. An ionization chamber is mounted on the detector holder, which is attached to a moving arm. The dimensions of the water tank are $60\times 60\times {50\;\text{cm}}^{3}$.

### B. Profile measurements

Profiles for several field sizes (5×5, 10×10, 15×15, 20×20, and $30\times 30{\;\text{cm}}^{2}$ at 100 cm source‐to‐axis distance (SAD)) were acquired with the Dynamic Phantom using 6 MV and 18 MV photon beams delivered by Varian 21EX and 2100C LINACs, respectively, with the gantry at 0°. Radiation fields for the photon beams were defined by collimator jaws instead of a MLC to reduce beam edge uncertainty to less than 1 mm. Two scanned depths (1.0 cm and 5.0 cm in Lucite for 6 MV, 2.9 cm and 5.0 cm in Lucite for 18 MV) were acquired for each field size. Corresponding scanned effective depths (1.1 cm and 5.7 cm in water for 6 MV, 3.3 cm and 5.7 cm in water for 18 MV) were acquired for each field size with the water scanner. Profiles of the same set of field sizes and depths for the 6 MV and 18 MV beams were acquired with Dynamic Phantom with the gantry angles at 90° and 270°.

To study the effect of gantry rotation on MLC‐shaped photon beams delivered by a Varian 21EX with 120 MLC, beam profiles in the direction along the MLC motion for field sizes of 5×5 and $10\times 10{\;\text{cm}}^{2}$ (at 100 cm SAD with retracted collimator jaws) were acquired with the Dynamic Phantom at 0°, 90°, and 270° gantry angles. Beam profiles for the same field sizes were also acquired with the water tank at a 0° gantry angle. Scans at two depths, 1.0 cm and 5.0 cm in Lucite, and 1.1 cm and 5.7 cm in water, were acquired for each field size and gantry angle. For each field size and depth, profiles along (*X*) and perpendicular to (*Y*) the MLC leaf direction were acquired at a 0° gantry angle in both the Dynamic Phantom and the water tank. Profiles in the *X* direction were acquired only for a 90° gantry angle, and in the *Y* direction only for a 270° gantry angle.

For electron beams delivered by a Varian 2100C LINAC with the energies of 6 MeV, 12 MeV, and 20 MeV, profiles of several field sizes (10×10, 15×15, and $20\times 20{\;\text{cm}}^{2}$ defined at 100 cm source‐to‐surface distance (SSD)) at a 0° gantry angle were acquired with the Dynamic Phantom. The field size of electron beams was defined by a trimmer bar positioned at 95 cm SSD. Two scanned depths (1.0 cm and 1.4 cm in Lucite for 6 MeV, 1.0 cm and 2.9 cm in Lucite for 12 MeV and 20 MeV) were acquired for each field size. Corresponding scanned effective depths (1.1 cm and 1.6 cm in water for 6 MeV, 1.1 cm and 3.3 cm in water for 12 MeV and 20 MeV) are acquired for each field size with the water‐tank scanner. Profiles with the same field sizes and depths for the 6 MeV, 12 MeV, and 20 MeV electron beams were acquired with the Dynamic Phantom with the gantry angles at 90° and 270°.

Measurements of photon and electron beams with both the water tank and the Dynamic Phantom were performed with a PTW Freiburg 0.1 cm^3^ sealed chamber and a 1 mm scanning interval at 100 cm SAD and 100 cm SSD, respectively. During scanning with the Dynamic Phantom, the scanner was placed directly on the treatment table and was carefully aligned with a level. The uncertainty on the LINAC gantry position was less than 1°.

Since the Lucite/water‐to‐air ratios of the average mass energy absorption coefficient, ${\left(\frac{{\overset{―}{\mu }}_{en}}{\rho }\right)}_{air}^{med}$, for the megavoltage photon beams do not vary as a function of depth, the distribution of measured ionization readings is proportional to the relative dose profiles of the photon beams. Therefore, measured ionization distributions were used to present measured dose profiles in this study. To assure that the spectral distribution and the fluence of primary and scattered photons at a certain scanned depth of Lucite is the same as that at a comparable depth in water (i.e., at the corresponding effective depth in water), the scaling factor^(4)^ $\left({\left[{\overset{―}{\mu }}_{en}\right]}_{water}^{Lucite}\right)$ is used to obtain the effective depth in water. This is adopted from AAPM TG21. Since $\left({\left[{\overset{―}{\mu }}_{en}\right]}_{water}^{Lucite}\right)$ is proportional to the electron density of Lucite with respect to water, the relative electron density (1.137) is used as the scaling factor to calculate the effective depth in water used for photon beam in this study.

Since the ionization‐to‐dose conversion when using an ionization chamber for electron beam dosimetry depends strongly upon the electron's energy through the effects of mass stopping‐power ratios and other perturbation factors,^(5)^ caution should be taken to apply the appropriate correction factors. However, at depths less than the depth of $I_{50}$ (the depth at which the ionization reading is at the 50% of its maximum reading), the energy of electrons at the same depth does not vary significantly, since the primary electrons contribute the most ionization. Therefore, the ionization readings were acquired at depths less than $I_{50}$ depth to examine the effect of gantry rotation on electron beam profiles. The formula $Z_{w}=Z_{Luc}\;\rho_{w}^{Luc}{\left(\frac{s}{\rho }\right)}_{w}^{Luc}$, based on the scaling factor by the HPA (Hospital Physicists' Association 1985), ^(6)^ is used to calculate the effective depth in water for electron beams. The $Z_{w}$ and $Z_{Luc}$ are the scanning depths of water and Lucite, $\rho_{w}^{Luc}$ is the ratio of physical densities of Lucite to water, and ${\left(\frac{s}{\rho }\right)}_{w}^{Luc}$ is the mass stopping power ratio of Lucite to water. For profiles scanned at depths close to $d_{\text{max}}$, the ${\left(\frac{s}{\rho }\right)}_{w}^{Luc}$ is approximately 1.0 with small variations. Therefore, the ratio of physical densities (1.1363 g/cm^3^) of Lucite to water is used for calculating effective depth in this study.

## III. RESULTS

### A. Profiles at 0° gantry angle

**Figure 2:**
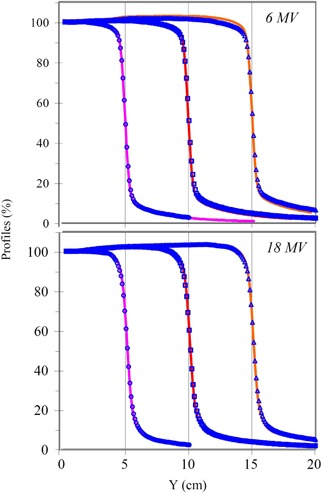
Profiles of field sizes (10 cm, 20 cm, and 30 cm) scanned at 5.0 cm depth in Lucite with the Dynamic Phantom (lines) and at 5.7 cm depth in water with water (symbols) are plotted for 6 MV and 18 MV photon beams in the top and bottom panels, respectively. All profiles were measured at a gantry angle of 0°.

**Figure 3:**
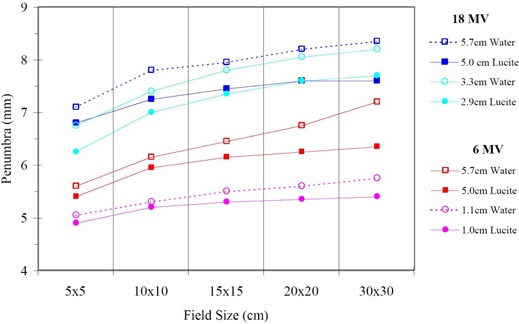
Extracted penumbra widths for profiles of 6 MV and 18 MV photon beams with the Dynamic Phantom (depths in Lucite) and water phantom (depths in water).

Figure 2 shows the comparisons between profiles acquired with the Dynamic Phantom at 5 cm depth in Lucite and with the water phantom at 5.7 cm depth in water with the gantry angle at 0° for 6 MV and 18 MV photon beams. The symmetry of each measured profile was within 1% for all fields acquired with the Dynamic Phantom and water tank. Results obtained by comparing the scanned profiles just inside of the field edges for both 6 MV and 18 MV at 5 cm depth of Lucite gave good agreement (<1% variance). The field edges of profiles scanned with the Dynamic Phantom and the water tank were aligned within 1 mm. Similar agreement was observed for 6 MV and 18 MV at a 1 cm depth in Lucite. However, lack of scattered radiation due to the finite size of the Dynamic Phantom produced lower doses outside the field edge and became more prominent (about 2%) for a $30\times 30{\;\text{cm}}^{2}$ field of a 6 MV beam. For a quantitative comparison, a summary of the measured penumbra widths for all measured profiles is listed in Table 1. The penumbra width is defined as the width between 20% and 80% of central axis dose. Penumbra widths range from 5 mm to 8 mm for the measured photon beam profiles. For 6 MV and 18 MV, penumbra widths of a given field size increase by about 0.5 mm as the depth increases. This increase of the penumbra width is a result of the divergence of primary beam. For a profile at a given depth in the same beam, about 1.0 mm increase in penumbra width is observed for an increase in field size from 5 cm to 30 cm. This increase is due to a larger number of scattered photons generated in a larger field. The penumbra widths at a given depth for the 18 MV beam are about 1.5 mm wider than those for the 6 MV beam. In addition, as shown in Fig. 3, the use of the Dynamic Phantom reduced the penumbra width by about 0.5 mm for both 6 MV and 18 MV beams in comparison with a standard water phantom scans.

**Table 1:** Penumbra widths of various field sizes and depths of scanned profiles for 6 MV and 18 MV photon beams at a 0° gantry angle with the Dynamic phantom and water phantom

	Depth (cm)	Field size (cm^2^)	5×5	10×10	15×15	20×20	30×30
Penumbra (mm)							
6 MV	1.0	Dynamic	4.9	5.2	5.3	5.35	5.4
	1.1	water	5.05	5.3	5.5	5.6	5.75
	5.0	Dynamic	5.4	5.95	6.15	6.25	6.35
	5.7	water	5.6	6.15	6.45	6.75	7.2
							
18 MV	2.9	Dynamic	6.25	7	7.35	7.6	7.7
	3.3	water	6.75	7.4	7.8	8.05	8.2
	5.0	Dynamic	6.8	7.25	7.45	7.6	7.6
	5.7	water	7.1	7.8	7.95	8.2	8.35

**Figure 4:**
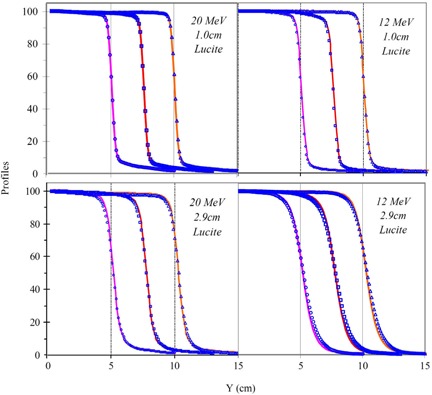
Left panels: Profiles of field sizes (10, 15 cm, and 20 cm) scanned with the Dynamic Phantom (lines) and with water (symbols) are plotted for the 20 MeV electron beam. Scanned depths at 1.0 cm and 2.9 cm in Lucite (i.e., 1.1 cm and 3.3 cm in water) are shown in the top and bottom panels, respectively. Right panels: Same field sizes and depths for the 12 MeV electron beam.

Due to the limited available thickness of Lucite slabs, profiles acquired at 1 cm and 2.9 cm depths in Lucite were used. Since the 2.9 cm depth in Lucite is deeper than the depth of $I_{50}$ for a 6 MeV electron beam, profiles of the 6 MeV beam were scanned at a 1.4 cm depth in Lucite instead of 2.9 cm to avoid the scanned depth deeper than $I_{50}$. The symmetry of each measured profile was within 2% for all fields and all energies acquired with the Dynamic Phantom and water phantom. Profiles of the 20 MeV beam at both 1.0 cm and 2.9 cm depths in Lucite as shown in Fig. 4 reveal good agreement (less than 2%) between the Dynamic Phantom and water phantom. Although similar good agreement between profiles scanned at a 1 cm depth in Lucite for the 12 MeV beam was observed, large disagreement (about 3%) was observed between profiles scanned at a 2.9 cm depth in Lucite as shown in Fig. 4. For a quantitative comparison, a summary of measured penumbra widths for all measured field sizes and electron energies is listed in Table 2. Penumbra widths fall between 5 mm and 15 mm for the measured profiles of electron beams. Penumbra widths increase with increasing depths due to multiple scattering. At the scanned 1.0 cm depth in Lucite, penumbra widths of profiles with the Dynamic Phantom agree to within 1 mm with those at effective depths with the water phantom. The penumbra increases with the depth for all field sizes and energies. The difference is more than 2 mm for 12 MeV electron beam penumbra measured at 2.9 cm depth in Lucite and at 3.3 cm depth in water.

**Table 2:** Penumbra widths for various field sizes and depths of scanned profiles for 6 MeV, 12 MeV, and 20 MeV electron beams at 0° gantry angle with the Dynamic Phantom and water phantom

	Depth (cm)	Field size (cm^2^)	5×5	10×10	20×20
Penumbra (mm)					
6 MeV	1.0	Dynamic	9	9.4	9.3
	1.1	water	9.7	10.2	10
	1.4	Dynamic	11.5	11.9	11.65
	1.6	water	12.5	13.1	12.85
					
12 MeV	1.0	Dynamic	5.45	5.7	5.5
	1.1	water	5.6	5.95	5.65
	2.9	Dynamic	13.15	13.5	13.3
	3.3	water	15.4	16.35	15.7
					
20 MeV	1.0	Dynamic	4.8	4.75	4.7
	1.1	water	4.85	4.9	4.8
	2.9	Dynamic	8.35	8.45	8.35
	3.3	water	9.8	9.8	9.75

### B. Profiles at various gantry angles

**Figure 5:**
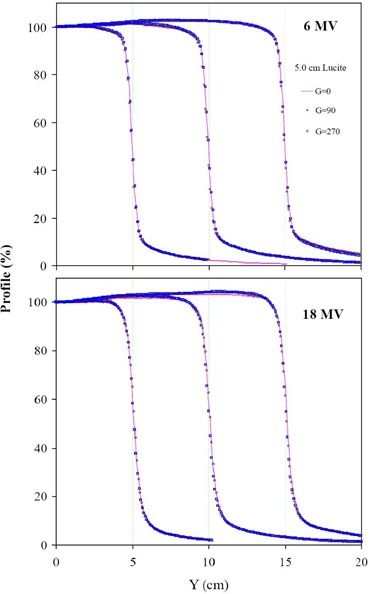
Profiles of field sizes (10 cm, 20 cm, and 30 cm) of collimator‐shaped photon beams scanned at a 5.0 cm depth in Lucite with the Dynamic Phantom at gantry angles of 0°, 90°, and 270° are plotted for 6 MV and 18 MV photon beams in the top and bottom panels, respectively.

Figure 5 shows profiles scanned at gantry angles of 0°, 90°, and 270° for 6 and 18 MV collimator‐shaped photon beams at a 5 cm depth in Lucite for a constancy check on the effect of gantry rotation. The symmetry of each measured profile was within 1% for all fields at gantry angles of 90° and 270°. General good agreement is seen (less than 1%) for all field sizes of 6 MV and 18 MV photon beams. Similar agreement was observed for profiles scanned at 1.0 cm depth in Lucite with 6 and 18 MV photon beams.

**Figure 6:**
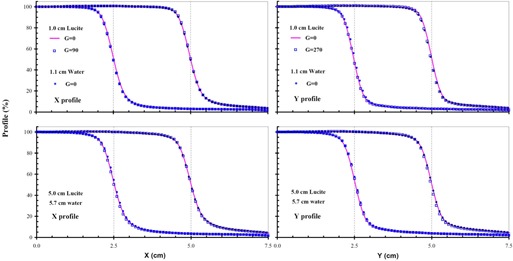
*X* and *Y* profiles of the field sizes (5 cm and 10 cm) for the MLC‐shaped 6 MV photon beams are plotted, respectively, in the left and right panels. Both the *X* and *Y* profiles at the gantry angle of 0° are plotted for 1 cm and 5 cm depths of Lucite with the Dynamic Phantom and 1.1 cm and 5.7 cm depth of water with the water tank. *X* profiles at the gantry angle of 90° and *Y* profiles at the gantry angle of 270° are also plotted. The *X* and *Y* are in the directions of along and perpendicular to the MLC leaf, respectively.

Figure 6 shows *X* (transverse) and *Y* (radial) profiles for the 6 MV MLC‐shaped photon beams. The symmetry of each measured profile was less than 1%. At a 0° gantry angle, *Y* profiles measured with the Dynamic Phantom for agreed with the profiles measured with the water tank for both scanning depths to within 2%. The *Y* profiles measured at a 270° gantry angle with the Dynamic Phantom also agreed with the profiles measured at a 0° gantry angle. A good agreement was observed between the *X* profiles measured with the Dynamic Phantom at gantry angles of 0° and 90°. Similar gantry angle variations for both *X* and *Y* profiles of the MLC‐shaped photon beams scanned using the Dynamic Phantom indicate that the uncertainty in the MLC position is similar to that for the collimator jaws.

**Figure 7:**
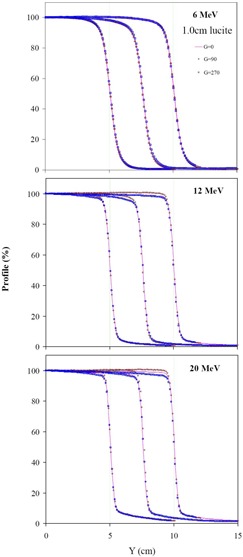
Profiles of field sizes (10 cm, 15 cm, and 20 cm) scanned at a 1.0 cm depth in Lucite with the Dynamic Phantom at gantry angles of 0°, 90°, and 270° are plotted for 6 MeV, 12 MeV, and 20 MeV electron beams.

Figure 7 shows profiles scanned at gantry angles of 0°, 90°, and 270° for 6 MeV, 12 MeV, and 20 MeV electron beams at 1.0 cm depth in Lucite for the effect of gantry rotation. The symmetry of each measured profile was within 1% for all fields at gantry angles of 90° and 270°. A difference between the 90 and 270 profiles of up to 4% was observed near the field edge for the 20′ 20 cm^2^ field of the 20 MeV electron beam. To investigate observed disagreement for profiles scanned at gantry angles of 0° and 90°/270° is beyond the scope of this paper. Similar results were obtained for profiles scanned at larger depths of 1.4 cm in Lucite for 6 MeV electron beam and of 2.9 cm in Lucite for the 12 MeV and 20 MeV electron beams.

## IV. Discussion and Conclusion

Using the Dynamic Phantom scanner for measuring profiles of electron beams produces large differences in measured penumbra widths with respect to a water phantom scanner. Since the scaling factor used to calculate effective depths does not take into account differences in the mass angular scattering power^(7,^, ^8)^ between Lucite and water, the observed differences in penumbra width may be mainly caused by this variation in angular scattering power. Therefore, the Dynamic Phantom scanner should not be used to measure primary electron beam profiles for dosimetry purposes.

Based on the good agreement between profiles with the Dynamic and water phantom for collimator‐shaped 6 MV and 18 MV at a 0° gantry angle, Dynamic Phantom scan measurements are acceptable for beam flatness and symmetry QA checks. Additional good results were obtained for profiles of a collimator‐shaped beam at different gantry angles scanned with the Dynamic Phantom scanner. Observed similar variations between profiles of a MLC‐shaped photon beam at gantry angles of 0°, 90°, and 270° indicate that the uncertainty in MLC position is similar to that for the collimator jaws. We conclude that the Dynamic Phantom scanner has the potential to be a useful device for the routine quality assurance on beam profiles of photon beams and constancy check of electron beams at various gantry angles.
